# Persistent Differences in Brain Structure in Developmental Dyscalculia: A Longitudinal Morphometry Study

**DOI:** 10.3389/fnhum.2020.00272

**Published:** 2020-07-17

**Authors:** Ursina McCaskey, Michael von Aster, Ruth O’Gorman, Karin Kucian

**Affiliations:** ^1^Center for MR-Research, University Children’s Hospital, Zurich, Switzerland; ^2^Children’s Research Center, University Children’s Hospital, Zurich, Switzerland; ^3^Clinic for Child and Adolescent Psychiatry, German Red Cross Hospitals, Berlin, Germany; ^4^Neuroscience Center Zurich, University of Zurich and ETH Zurich, Zurich, Switzerland; ^5^Zurich Center for Integrative Human Physiology, University of Zurich, Zurich, Switzerland

**Keywords:** developmental dyscalculia, longitudinal, gray matter, white matter, children, development, voxel-based morphometry

## Abstract

Developmental dyscalculia (DD) is a learning disability affecting the acquisition of numerical-arithmetical skills. Affected people show persistent deficits in number processing, which are associated with aberrant brain activation and structure. Reduced gray matter has been reported in DD for the parietal cortex including the intraparietal sulcus (IPS), but also the frontal and occipito-temporal cortex. Furthermore, dyscalculics show white matter differences for instance in the inferior (ILF) and superior longitudinal fasciculus (SLF). However, the longitudinal development of these structural differences is unknown. Therefore, our goal was to investigate the developmental trajectory of gray and white matter in children with and without DD. In this longitudinal study, neuropsychological measures and T1-weighted structural images were collected twice with an interval of 4 years from 13 children with DD (8.2–10.4 years) and 10 typically developing (TD) children (8.0–10.4 years). Voxel-wise estimation of gray and white matter volumes was assessed using voxel-based morphometry for longitudinal data. The present findings reveal for the first time that DD children show persistently reduced gray and white matter volumes over development. Reduced gray matter was found in the bilateral inferior parietal lobes including the IPS, supramarginal gyri, left precuneus, cuneus, right superior occipital gyrus, bilateral inferior and middle temporal gyri, and insula. White matter volumes were reduced in the bilateral ILF and SLF, inferior fronto-occipital fasciculus (IFOF), corticospinal tracts, and right anterior thalamic radiation (ATR). Behaviorally, children with DD performed significantly worse in various numerical tasks at baseline and follow-up, corroborating persistent deficits in number processing. The present results are in line with the literature showing that children with DD have reduced gray and white matter volumes in the numerical network. Our study further sheds light on the trajectory of brain development, revealing that these known structural differences in the long association fibers and the adjacent regions of the temporal- and frontoparietal cortex persist in dyscalculic children from childhood into adolescence. In conclusion, our results underscore that DD is a persistent learning disorder accompanied by deficits in number processing and reduced gray and white matter volumes in number related brain areas.

## Introduction

Numbers and mathematics are omnipresent in our daily lives and their mastery is crucial to function effectively in our society. Poor numeracy skills, therefore, pose a serious burden for persons affected. Developmental dyscalculia (DD) is a learning disorder characterized by significant and persisting difficulties in learning academic skills related to mathematics or arithmetic. The difficulties are not due to a disorder of intellectual development, sensory impairment, mental or neurological disorders, or inadequate instruction (WHO, 2018). DD affects about 3–7% of the school children (Wyschkon et al., 2009; Butterworth et al., 2011) and has been shown to have a persisting character (Shalev et al., 2005; Geary et al., 2013; McCaskey et al., 2018). Studies in children with DD reveal impairments in numerical magnitude processing and difficulties in the retrieval of arithmetical facts from memory, but also in visuospatial memory or inhibition (Geary, 1993; Landerl et al., 2004; De Smedt et al., 2013; Szucs et al., 2013). These deficiencies have been linked to abnormalities in brain function and structure. When processing numbers and performing arithmetic, a large neural network is involved including posterior parietal (intraparietal sulcus (IPS), angular gyrus, supramarginal gyrus), prefrontal, occipito-temporal and hippocampal areas. Children with DD show aberrant activation of the numerical neural network (Price et al., 2007; Davis et al., 2009; Kucian et al., 2011; Ashkenazi et al., 2012) and abnormalities in different measures of brain structure (e.g., fractional anisotropy, cortical thickness, cortical surface area, gray and white brain volumes; Rykhlevskaia et al., 2009; Kucian et al., 2013; Ranpura et al., 2013).

Hitherto, few cross-sectional studies investigated structural differences in white and gray matter volumes in children with DD compared to typically developing (TD) peers (Rotzer et al., 2008; Rykhlevskaia et al., 2009; Ranpura et al., 2013). Generally, all studies report reduced gray matter volumes in dyscalculics in the IPS and the inferior and superior parietal lobes (Rotzer et al., 2008; Rykhlevskaia et al., 2009; Ranpura et al., 2013). These regions have been linked to number processing and mathematical problem-solving in several studies (Dehaene et al., 2003; for a meta-analysis see Arsalidou et al., 2018). Furthermore, decreased volumes are detected in regions of the frontal lobe such as the anterior cingulum and the inferior and middle frontal gyrus (Rotzer et al., 2008), known to be involved in working memory, attention and goal-directed behavior (Arsalidou et al., 2018), and in occipital regions such as the cuneus/precuneus, lateral occipital cortex, lingual and fusiform gyrus (Rykhlevskaia et al., 2009), which process visual numerical information. Finally, less gray matter volume is also found in the entorhinal cortex, the parahippocampal gyrus and the hippocampus (Rykhlevskaia et al., 2009; Ranpura et al., 2013), which is suggested to play an important role in the formation of long-term memory for arithmetical facts (Menon, 2016). In contrast to the results in children, the findings in studies with dyscalculic adults are less clear. A recent study found no differences in volumetric or surface characteristics of gray matter in dyscalculic adults with and without comorbid dyslexia compared to a control group (Moreau et al., 2019). Likewise, Cappelletti and Price (2013) did not find differences in gray matter volume in a whole-brain analysis in adults with DD but could show that the right parietal area had significantly reduced gray matter volume in a region of interest (ROI) analysis. However, a study investigating the structural correlates of mathematical expertise revealed higher gray matter volume in the right superior parietal lobe, but lower gray matter volume in the right IPS in professional mathematicians compared to non-mathematicians (Popescu et al., 2019).

Differences between children with and without DD are not only found for gray matter structures since white matter volume has also been reported to be reduced in children with DD. Less white matter is observed in temporoparietal regions (right inferior parietal lobe, temporal pole, transverse temporal lobe) and the left frontal lobe (Rotzer et al., 2008; Rykhlevskaia et al., 2009; Ranpura et al., 2013). These regions are part of prominent white matter tracts. The inferior (ILF) and superior longitudinal fasciculus (SLF) have been suggested to be particularly important for numerical processing, as they may be involved in frontoparietal communication and visual processing of numerical or mathematical problems (van Eimeren et al., 2008; Tsang et al., 2009; Matejko and Ansari, 2015). Further white matter differences are found in regions that correspond to the inferior fronto-occipital fasciculus (IFOF), forceps major, corticospinal tract (CST), and the anterior thalamic radiation (ATR). Interestingly, fractional anisotropy (FA), a measure for white matter integrity, is reduced in the SFL, ILF, IFOF and the caudal forceps major in children with DD (Rykhlevskaia et al., 2009; Kucian et al., 2013; for a review see Matejko and Ansari, 2015). Note that no studies to date have reported increased gray or white matter volumes for persons with DD compared to peers without DD (Rotzer et al., 2008; Rykhlevskaia et al., 2009; Cappelletti and Price, 2013; Ranpura et al., 2013; Moreau et al., 2019).

Currently, there is one study that investigated children between 8 and 14 years and aimed to describe how the differences between dyscalculic and control children vary during cortical development (Ranpura et al., 2013). Relative to TD children, gray matter volume in dyscalculics increases with age in the left dorsolateral prefrontal cortex and the right superior occipital lobe, but decreases slightly in the left primary motor cortex. White matter development of children with DD showed notable delays relative to the control group. Whilst TD children showed an age-related increase in frontal and parietal areas, the white matter volumes of DD children remained stable over time (Ranpura et al., 2013).

The gray and white matter regions and their relationship with numerical or arithmetical skills were also investigated in TD children. Gray matter volume is positively correlated with arithmetic scores or performance gains specifically in the left IPS and angular gyrus (Li et al., 2013; Supekar et al., 2013; Evans et al., 2015). Price et al. (2016) showed that gray matter volume in the left IPS measured in first grade predicted the math score at the end of the second grade. This result is not confined to the parietal cortex. Increased gray matter volume in the frontal (dorsal and ventral prefrontal cortices, IFG), occipito-temporal (cuneus, fusiform gyrus) and in the hippocampus also relates to better math performance (Li et al., 2013; Supekar et al., 2013; Evans et al., 2015; Wilkey et al., 2018). Moreover, associations between brain volume abnormalities and math performance have been reported for the gray matter volume of parietal regions in other populations prone to math difficulties (prematurely born children, very low birth weight, Turner syndrome; Isaacs et al., 2001; Starke et al., 2013; Zhao et al., 2013).

To summarize, gray matter volume in various regions of the frontoparietal network, but specifically in the IPS—which is known as a key area for number processing—has been associated with better performance in numerical processing and arithmetical skills. In line with that, children with DD show reduced gray matter volumes in parietal, but also frontal, occipito-temporal and hippocampal areas. Furthermore, white matter differences have been reported in the main white matter tracts connecting the parietal with the frontal and the temporal cortex in subjects with DD. However, to the author’s best knowledge, there is no study investigating the development of these structural differences in children with DD using a longitudinal study design. Therefore, the present work aims to elucidate the developmental trajectory of gray and white brain matter volume in children with and without DD from childhood to adolescence.

Based on previous literature, we expect to replicate the known group differences. Reduced gray matter volumes in various regions of the frontoparietal numerical network are expected in children with DD compared to an age-matched control group with normal math performance (Rotzer et al., 2008; Rykhlevskaia et al., 2009; Ranpura et al., 2013). Furthermore, we hypothesize that less white matter volume will be present in the main tracts connecting the parietal with the frontal and the temporal lobe for dyscalculic children (Rotzer et al., 2008; Rykhlevskaia et al., 2009). Second, we will examine the general developmental effects of white and gray matter substance. We anticipate an increase in white matter and possibly a decrease in the gray matter over the examined time from childhood to early adolescence. Longitudinal studies focusing on the structural development from childhood to adulthood showed that gray matter volume increases in the first 10 years of life followed by a decrease in the next decades (Mills et al., 2016). However, the peak of the gray matter volume varies though between studies and also brain regions (Giedd et al., 1999; Gogtay and Thompson, 2010; Groeschel et al., 2010; Mills et al., 2016). Findings regarding white matter volumes revealed a constant increase from childhood until young adulthood (Giedd et al., 1999; Groeschel et al., 2010; Mills et al., 2016). Finally, we are interested in the longitudinal trajectory of the group differences. According to the results of Ranpura et al. (2013), we expect a relative increase in gray matter volume and no change in white matter volume over time in children with DD compared to TD peers. In contrast, the findings in adults point towards a normalization of the gray matter structure over time, as no or only little differences were found in dyscalculic adults. Based on this literature we, therefore, expect to find divergent developmental trajectories in dyscalculic compared to controls.

## Materials and Methods

### Participants and Procedure

A total of 35 (23 DD, 12 TD) children between 8 and 11 years were recruited into this longitudinal study, of which 27 took part in a previous study (Kucian et al., 2011). This longitudinal study included structural and functional MRI data (for the results of fMRI data please see McCaskey et al., 2018). Children were evaluated by neuropsychological tests and MRI at baseline and returned after 4.2 (*SD* = 0.46) years for a follow-up measurement. We approached the subjects of our study through the school setting or School psychological Services (DD subjects). The children visited us twice at the Center for MR-Research of the University Children’s Hospital Zurich. On both occasions, they first completed a neuropsychological session and then underwent the MRI measurement.

Inclusion criteria for all children were no history of neurological or psychiatric disorders and an IQ ≥ 85, measured by the third edition of the WISC (Tewes et al., 1999; Similarities, Block Design, Vocabulary, Picture Arrangement). Furthermore, DD children had to score below the 10th percentile in the total score or three subtests of a standardized numerical test battery (ZAREKI-R) at baseline. These criteria are in line with the diagnostic criteria for DD of the ICD-11 (6A03.2 Developmental learning disorder with impairment in mathematics; WHO, 2018). TD children had to perform above the cut-off of value in the test batteries for numerical abilities at baseline and follow-up (10th percentile in the ZAREKI-R and 67 points in the BASIS-MATH 4–8, respectively). Following these criteria, six DD children and one TD child were excluded from the study. For the MRI analysis, two children were excluded due to missing imaging data at baseline or follow-up and three for image quality reasons, resulting in 13 DD and 10 TD complete data sets for the whole study. Groups did not differ in age, gender, handedness, and pubertal status determined by the Edinburgh Handedness Inventory (Oldfield, 1971) and an adapted version of the Self-administered Rating Scale for Pubertal Development (Carskadon and Acebo, 1993).

Parental consent and child assent were obtained at the beginning of the study. The study was approved by the Ethics committee of Zurich, Switzerland based on guidelines from the World Medical Association’s Declaration of Helsinki (WMA, 2013).

### Neuropsychological Testing

During the neuropsychological assessment, we acquired numerical abilities, general cognitive abilities as well as measures for the most common comorbid disorders such as developmental dyslexia, attention deficit and hyperactivity disorder, and working memory deficits.

#### Numerical Abilities

At baseline, numerical abilities were assessed using the revised version of the Neuropsychological Test Battery for Number Processing and Calculation in Children (ZAREKI-R; von Aster et al., 2006). The Zareki-R is a multidimensional test, measuring basic numerical skills as well as calculation, and widely used for the diagnosis of DD in the German-speaking area (see Supplementary Material for detailed information about the subtests). Based on this test battery children scoring below the 10th percentile in the total score or three subtests were identified with DD.

Also, the Arithmetic subtest of the Wechsler Intelligence Scale for Children (WISC-III; Tewes et al., 1999) was performed. In this subtest, children had to solve story problems of increasing difficulty within a set time limit (reported test values are IQ scores).

At follow-up, the numerical achievement was assessed with the test for Basic Diagnosis in Mathematics Education for Grades 4-8 (BASIS-MATH 4-8; Moser Opitz et al., 2010). The Basis-Math is a criterion-based test battery measuring various arithmetical abilities such as counting, decimal system, and calculation. Criteria for numerical deficiencies are met if the performance is under a threshold value of 67 points (maximum score of 83 points). This is interpreted as not reaching mastery of basic mathematical concepts (see Supplementary Material for detailed information).

The curriculum-based subtest Quantity Comparison of the Cognitive Abilities Test (KFT 4-12+R; Heller and Perleth, 2000) was performed to assess the arithmetic performance at a peer level. In this subtest, subjects had 10 min to solve as many quantity comparisons as possible of increasing difficulty (reported test values are T scores).

The spatial representation of numbers was measured using a computerized number line task adopted from Kucian et al. (2011; for a detailed description see McCaskey et al., 2018). Children had to indicate by mouse-click the position of 20 Arabic digits on a number line with the labeled endpoints 0 and 100. Accuracy was measured by calculating the percentage distance from the marked to the correct position of the given number (reported measures are raw values).

Children also solved 40 basic arithmetic problems (20 addition and 20 subtraction) in the number range 0–1,000 (for a detailed description see McCaskey et al., 2018). Each problem was presented visually on the computer screen and solutions were given *via* the keyboard. The number of correctly solved items was quantified (reported test values are raw scores, maximum value 20).

#### Domain General Cognitive Abilities

At baseline, intelligence was measured with the third edition of the WISC (Similarities, Block Design, Vocabulary, Picture Arrangement; Tewes et al., 1999). At follow-up, the fourth edition of the WISC was used (Similarities, Block Design, Matrix Reasoning; Petermann and Petermann, 2007). Table 1 shows the estimated general IQ.

**Table 1:** Demographic characteristics and scores on numerical abilities, domain-general cognitive abilities, working memory, attention, and reading.

Behavioral measure	DD		TD		Test-statistic	*p*	*r*
	*N*	*M (SD)*	*N*	*M (SD)*			
**Baseline assessment**							
Age	13	9.5 (0.7)	10	9.2 (0.8)	56.0^a^	0.605	0.13
Gender m/f	13	3/10	10	5/5		0.221	
Handedness l/a/r	13	1/4/8	10	1/3/6	0.04^b^	0.999	
**Numerical abilities**							
DD diagnosis *(ZAREKI-R)*	13	6.3 (5.0)	10	75.6 (19.5)	0.00^a^	0.000***	0.84
Arithmetic *(WISC-III)*	12	90.4 (9.6)	10	105.5 (12.8)	21.5^a^	0.008**	0.55
**Domain general cognitive abilities**							
Estimated IQ *(WISC-III)*	13	99.8 (5.8)	10	111.6 (6.9)	11.5^a^	0.000***	0.69
**Working memory**							
Visuo-spatial *(BST)*	11	2.7 (1.5)	10	3.6 (1.0)	37.5^a^	0.195	0.31
**Follow-up assessment**							
Age	13	13.5 (0.9)	10	13.6 (0.8)	64.0^a^	0.976	0.01
Puberty Score	13	2.8 (0.7)	10	2.6 (0.8)	57.0^a^	0.636	0.10
**Numerical abilities**							
DD diagnosis *(BASIS-MATH 4-8)*	13	49.8 (9.1)	10	75.3 (4.2)	0.00^a^	0.000***	0.84
Quantity Comparison *(KFT 4-12+R)*	11	41.4 (3.7)	10	53.4 (4.5)	1.0^a^	0.000***	0.83
Number line task (% distance)	13	5.3 (1.9)	10	3.6 (2.2)	18.0^a^	0.002**	0.61
Addition (accuracy)	13	15.9 (4.0)	10	18.6 (1.4)	25.5^a^	0.010*	0.52
Subtraction (accuracy)	12	12.5 (4.1)	10	17.6 (2.5)	16.0^a^	0.002**	0.62
**Domain general cognitive abilities**							
Estimated IQ *(WISC-IV)*	13	100.5 (6.9)	10	113.0 (5.7)	9.5^a^	0.000***	0.72
**Working memory**							
Visuo-spatial *(BST)*	13	5.8 (1.8)	10	6.8 (2.0)	46.5^a^	0.243	0.25
Verbal *(WISC-IV)*	13	98.9 (12.1)	10	107.5 (9.2)	36.5^a^	0.074	0.37
**Attention**							
Alertness *(TAP)*	13	47.3 (11.5)	10	46.5 (10.4)	57.5^a^	0.659	0.10
Go-Nogo *(TAP)*	12	56.8 (32.9)	10	63.5 (24.1)	54.0^a^	0.710	0.08
**Reading**							
Words *(SLRT-II)*	12	19.1 (19.3)	10	12.9 (11.7)	55.5^a^	0.783	0.06
Pseudowords *(SLRT-II)*	11	24.5 (18.9)	10	19.3 (13.1)	45.0^a^	0.498	0.15

ZAREKI-R, Neuropsychological Test Battery for Number Processing and Calculation in Children (PR); BASIS-MATH 4-8, Basic Diagnostic in Mathematics for Grades 4-8 (raw score); WISC, Wechsler Intelligence Scale for Children (IQ score); KFT 4-12+R, Cognitive Abilities Test (T score); BST, Block-Suppression-Test (raw score); TAP, Testbattery for Attentional Performance (PR); SLRT-II, Salzburg Reading and Orthography Test (PR). ^a^Mann–Whitney U Test, ^b^Fisher’s Exact Test. *p < 0.05, **p < 0.01, ***p < 0.001.

#### Working Memory

Visuospatial working memory was measured with the Block-Suppression-Test (Beblo et al., 2004). The task required subjects to reproduce every second block of a previously presented sequence on a board with nine cubes. The sequences had a length of 3–9 cubes. Three items per sequence were presented. The longest sequence which was reproduced correctly twice was quantified (reported test values are raw scores, maximum value 9). Verbal working memory was measured with the subtest Digit Span of the WISC-IV (Petermann and Petermann, 2007). In this task, subjects had to repeat an auditorily presented sequence of numerals forward or backward. The sequences had a length of 2–9 numerals (reported test values are IQ scores).

#### Attention

Levels of attention and inhibition were measured using the subtests Alertness and Go-Nogo of the computerized Test battery for Attentional Performance (TAP; Zimmermann and Fimm, 1993). In the Alertness subtest, subjects had to react as quickly as possible when the target stimulus “x” appeared (intrinsic alertness). Half of the trials were preceded by an acoustic cue stimulus (phasic alertness). The test has four runs and a total of 80 target items. For each subject, the percentile rank of the median RT was quantified (reported test values are percentile ranks). In the Go-Nogo subtest, subjects had to react as quickly as possible to a target stimulus (“x,” go condition), but inhibit reactions on a second presented stimulus (“+,” nogo condition). The test has a total of 40 items (20 go and 20 nogo items). For each subject, the percentile rank of the median RT was quantified (reported test values are percentile ranks).

#### Reading Abilities

The 1-Minute-Reading-Task from the Salzburg Reading and Orthography Test (SLRT-II; Moll and Landerl, 2010) assessing word and pseudoword reading fluency was used to estimate the reading performance. Two sheets of paper with either 156 words or 156 pseudowords of increasing length and difficulty were presented. Subjects had 1 min per sheet to read as many words as possible. The amount of correctly read items was quantified (reported test values are percentile ranks). Because of lacking test norms in grades 7 and 8, we interpolated the norms from the test manual (grade 6) and Kronschnabel et al. (2013; grade 9).

#### Behavioral Data Analysis

Behavioral data were statistically analyzed with SPSS (Version 22). To account for the difficulties regarding the performance of statistical tests of normality in small samples, we performed nonparametric tests (Mann–Whitney *U* Test) to assess group differences. Effect sizes are reported as Pearson’s correlation coefficient *r* and are interpreted as small (*r* = 0.10), medium (*r* = 0.30) or large (*r* = 0.50).

### Brain Imaging

#### Image Acquisition

MRI data were acquired on a 3T General Electric Signa Scanner (GE Medical Systems, USA) using an 8-channel head coil. T1-weighted structural images (voxel size = 0.94 × 0.94 × 1.00 mm^3^) were acquired with a fast spoiled gradient echo sequence (3D FSPGR, slice thickness = 1 mm, no interslice skip, matrix size = 256 × 256, field of view = 240 mm, flip angle = 20°, echo time = 3 ms, repetition time = 10 ms). Participants were carefully instructed and supplied with hearing protection before entering the scanner. To minimize head motion, the head was stabilized with padding.

#### MRI Data Preprocessing

The data were preprocessed using the Computational Anatomy Toolbox (CAT12, Structural Brain Mapping Group, University of Jena, Germany), which is an extension to Statistical Parametric Mapping (SPM 12, Wellcome Trust Centre for Neuroimaging, University College London, UK) running on Matlab (Release 2012b, The MathWorks Inc., USA).

In a first step, the longitudinal data pair (baseline and follow-up image) of each subject was registered to the mean image for each subject by an inverse-consistent realignment, which also includes a bias correction between the different time points. The mean image of each subject is then segmented and the spatial normalization parameters are estimated with the help of a Dartel Normalization. These spatial normalization parameters (Dartel deformations) are then applied to the gray and the white matter segmentations of the baseline and follow-up image. The white and gray matter segmented images were smoothed with a Gaussian kernel of 8 mm FWHM (full width half maximum). As the last step, the total intracranial volume (TIV) was estimated for the baseline and follow-up image of each subject (please see Supplementary Material for the Matlab scripts of the preprocessing steps).

#### Quality Control

The CAT12 Toolbox provides image quality measures describing the properties of the images before the preprocessing. The image quality rating (IQR) is a weighted average of the noise contrast ratio, the inhomogeneity contrast ratio and the resolution of the input image. The images all reached ratings above 79 at both measurement time points (baseline range 79–87, follow-up range 81–87). Note that typical scientific (clinical) data is expected to get good to satisfactory ratings (70–90; Gaser and Dahnke, 2016, see also www.neuro.uni-jena.de/cat/). The groups did not differ in their IQR at baseline (DD *Mdn* = 85.0, TD *Mdn* = 85.3, *U* = 46.5, *z* = −1.15, *p* = 0.263) or follow-up (DD *Mdn* = 85.0, TD *Mdn* = 85.5, *U* = 48.0, *z* = −1.05, *p* = 0.313), and there was no significant difference in the quality measures between the time points (baseline *Mdn* = 85.1, follow-up *Mdn* = 85.3, *z* = −0.69, *p* = 0.494).

Also, the segmented and normalized gray and white matter images were visually inspected and the sample was checked for homogeneity (mean correlation). Based on the visual inspection and the Mahalanobis distance, which combines a measure of image quality before (weighted overall image quality) and after preprocessing (mean correlation), we excluded two DD and one TD data sets resulting in the final group size of 13 DD and 10 TD.

### Statistical Model

For the statistical analyses of the gray and white matter volumes, two separate flexible factorial models with the factors subject, group (DD, TD) and time (baseline, follow-up) including TIV and puberty score as covariates were defined. Statistical results are shown with a threshold of *p* < 0.05 family-wise error (FWE) correction (see Supplementary Material for the design matrix and the Matlab scripts of the statistical model as well as the defined contrasts). Anatomical localization of the gray matter volume results was attained trough the SPM Anatomy Toolbox v2.0 (Eickhoff et al., 2005, 2007). White matter regions were labeled according to the JHU (Johns Hopkins University) white-matter tractography atlas (Hua et al., 2008).

## Results

### Behavioral Data

The neuropsychological results and the demographic data for all subjects are summarized in Table 1. All participants scored in the normal range of IQ (DD IQ = 93–111, TD IQ = 101–125). However, groups differed in the estimated general IQ at baseline (WISC-III *p* < 0.001, *r* = 0.69) and follow-up (WISC-IV *p* < 0.001, *r* = 0.72). IQ measures are known not to be fully independent of measures of math ability, and the present sample, therefore, reflects the cognitive pattern typically observed in DD.

In the attention, working memory, and reading task no differences between DD and TD children were found (all *p* > 0.05).

#### Numerical Achievement

As expected, numerical abilities, assessed by the Zareki-R at baseline, differed significantly between the TD and the DD groups (*p* < 0.001, *r* = 0.84). The groups also differed in the subtest Arithmetic (WISC-II), with the DD subjects scoring significantly lower than the TD group (*p* < 0.01, *r* = 0.55; Table 1).

At follow-up, adolescents of the DD group still performed worse in comparison to their peers (Basis-Math *p* < 0.001, *r* = 0.84). In fact, all the subjects identified with DD at baseline still fulfilled the diagnostic criteria for DD at the follow-up measurement. Furthermore, they also scored significantly lower in the curriculum based test Quantity Comparison (KFT 4-12+R *p* < 0.001, *r* = 0.83), the number line task (*p* < 0.01, *r* = 0.61) and the basic arithmetic operations (addition *p* < 0.05, *r* = 0.52, subtraction *p* < 0.01, *r* = 0.62; Table 1).

Pearson’s correlations of the whole brain gray and white matter volume with behavioral measures were calculated for baseline and follow-up. At the follow-up, gray matter volume was positively correlated with the Basis-Math (*r* =* 0*.64, *p* < 0.05) in TD children. However, we found a negative relationship in TD adolescents between performance in addition and gray (*r* = −0.69, both *p* < 0.05) and white matter volume (*r* = −0.60, *p* < 0.05), respectively. For DD children, no significant correlations between numerical abilities and volume of the brain structure were revealed (see Supplementary Material for a complete table of correlations).

### Structural Results

#### Gray Matter

**Figure 1:**
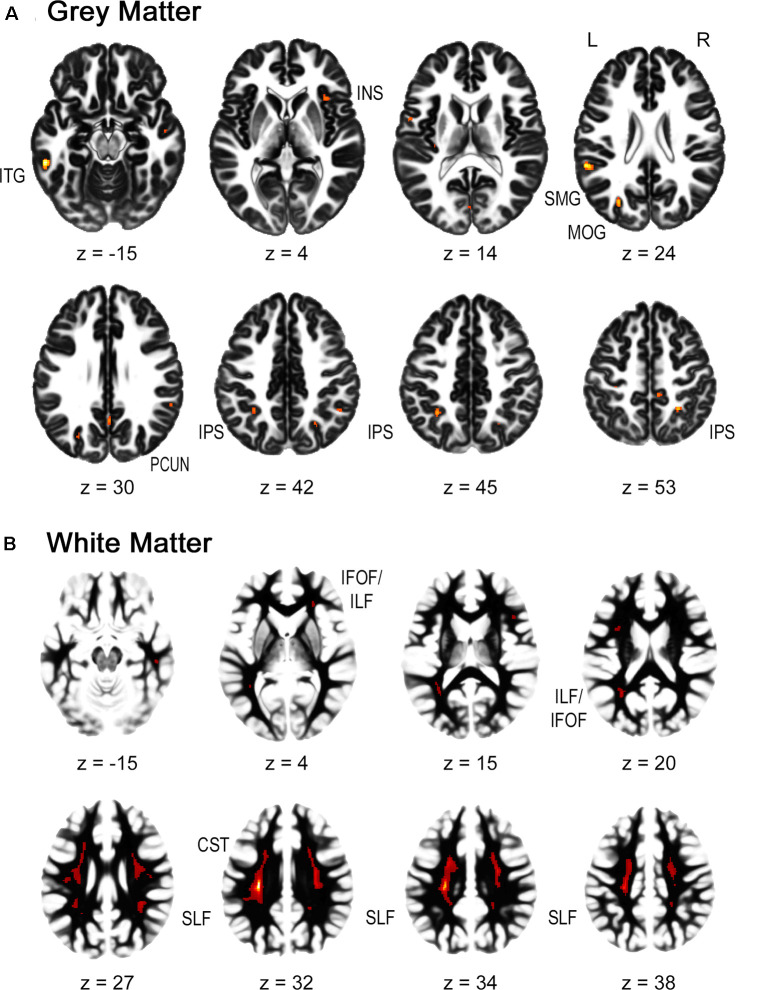
Group differences in gray and white matter. Results are shown on a pediatric template (Fonov et al., 2009) with a significance level of *p*-cluster < 0.05, FWE corrected. **(A)** Decreased gray matter volumes in dyscalculics compared to typically developing children over both time points. **(B)** Decreased white matter volumes in dyscalculics compared to typically developing children over both time points. Abbreviations: CST, corticospinal tract; IFOF, inferior fronto-occipital fasciculus; ILF, inferior longitudinal fasciculus; INS, insula; IPS, intraparietal sulcus; ITG, inferior temporal gyrus; MOG, middle occipital gyrus; PCUN, precuneus; SLF, superior longitudinal fasciculus; SMG, supramarginal gyrus.

For the gray matter volume, the flexible factorial analysis revealed a significant effect of group (Figure 1A, Table 2). DD children showed decreased gray matter volumes in the bilateral inferior parietal lobe assigned to the IPS, the bilateral supramarginal gyri, the left precuneus, the left postcentral gyrus, and the right paracentral lobule compared to TD children. In the occipital lobe, differences were found in the left calcarine gyrus/cuneus, the left middle occipital gyrus (MOG), and the right superior occipital gyrus (SOG). Decreased gray matter was found in the bilateral inferior (ITG) and middle temporal gyri (MTG), the left rolandic operculum, and the bilateral insula over the examined time of 4 years.

**Table 2:** Gray matter.

Region	Cluster size	*p*-corrected	MNI coordinates			
			*Z*-value	*x*	*y*	*z*
L middle occipital gyrus	106	<0.001	6.04	−28	−75	27
L inferior temporal gyrus	101	<0.001	6.52	−58	−39	−15
L supramarginal gyrus/inferior parietal lobe	96	<0.001	6.18	−58	−40	24
L intraparietal sulcus/inferior parietal lobe	39	0.002	5.61	−32	−52	45
L precuneus	35	0.006	5.41	2	−58	30
R insula	31	0.008	5.35	36	20	4
L calcarine gyrus, cuneus	30	0.022	5.12	0	−82	9
L middle temporal gyrus/inferior parietal lobe	25	0.002	5.61	−56	−62	8
R paracentral lobe/superior parietal lobe	24	0.008	5.35	9	−36	51
R superior parietal lobe/intraparietal sulcus	23	<0.001	6.02	27	−51	54
R intraparietal sulcus/inferior parietal lobe	18	0.013	5.25	48	−50	40
R supramarginal gyrus/inferior parietal lobe	17	0.007	5.39	58	−45	28
L rolandic operculum	17	0.012	5.26	−56	2	14
R superior occipital gyrus	11	0.019	5.15	26	−63	44
R inferior temporal gyrus	9	0.024	5.10	44	−52	−10
R middle temporal gyrus	7	0.020	5.14	52	−9	−15
L postcentral gyrus	6	0.008	5.34	−32	−32	54
R insula	5	0.018	5.16	36	8	8
R middle temporal gyrus	4	0.039	4.98	42	−63	0
L insula	3	0.035	5.01	−33	−24	15
R insula	2	0.032	5.03	40	8	−3
L supramarginal gyrus/inferior parietal lobe	1	0.044	4.95	−58	−22	21

*Peak coordinates and details of clusters from the whole brain voxel-based analyses (*p* < 0.05, FWE corrected)*.

The main effect of time and the interaction group by time were not significant.

#### White Matter

For the white matter volume, the flexible factorial analysis revealed a significant effect of the group (Figure 1B, Table 3). DD children showed reduced white matter volumes in a widespread set of brain regions including the bilateral CST, the bilateral superior and ILF, the bilateral IFOF, and the right ATR. Compared to TD children the DD children further showed increased white matter volumes in the right CST.

**Table 3:** White matter.

Region	Cluster size	*p*-corrected	MNI coordinates			
			*Z*-value	*x*	*y*	*z*
L corticospinal tract	1661	<0.001	>8.00	−28	−21	32
L superior longitudinal fasciculus		<0.001	>8.00	−21	−2	36
L superior longitudinal fasciculus		<0.001	7.46	−32	−9	28
R superior longitudinal fasciculus	677	<0.001	>8.00	27	−8	34
R superior longitudinal fasciculus		<0.001	>8.00	30	−21	32
N/A		<0.001	7.41	22	2	36
R superior longitudinal fasciculus	109	<0.001	7.39	34	−40	27
R anterior thalamic radiation	37	<0.001	6.54	22	−44	33
R superior longitudinal fasciculus	27	0.001	5.70	50	−27	−15
R corticospinal tract	25	0.008	5.23	16	−15	56
L inferior fronto-occipital fasciculus	22	0.005	5.34	−24	−84	−6
R inferior fronto-occipital fasciculus	17	0.009	5.20	26	33	4
R inferior longitudinal fasciculus/inferior fronto-occipital fasciculus	15	0.001	5.74	33	−68	9
N/A	9	0.002	5.48	44	20	15
L superior longitudinal fasciculus	2	0.039	4.84	−20	−46	51
L inferior longitudinal fasciculus	2	0.015	5.08	−46	−14	−21
L superior longitudinal fasciculus	1	0.046	4.80	−44	−28	28
N/A	1	0.045	4.81	34	−78	14
L inferior longitudinal fasciculus	1	0.043	4.82	−32	−80	−4
R inferior longitudinal fasciculus	1	0.040	4.84	46	−40	−8

*Peak coordinates and details of clusters from the whole brain voxel-based analyses (*p* < 0.05, FWE corrected)*.

The main effect of time revealed an increase in the left SLF adjacent to the precentral gyrus over both groups. The interaction group by time was not significant.

## Discussion

Our study aimed to investigate the neural structural development of children with DD and TD peers using a longitudinal study. Until now, there have only been a handful of studies investigating the structural differences between children with and without DD, and only one study examining changes of regional differences in cortical development (Rotzer et al., 2008; Rykhlevskaia et al., 2009; Ranpura et al., 2013). However, all of these study results are based on cross-sectional data. To our knowledge, this is the first study investigating the neural developmental trajectory using longitudinal data in children with DD. On the behavioral level, we found that the children of the DD group performed significantly worse in all the numerical and arithmetical tasks. This result remained stable over time. All children that were identified with DD at the beginning of the study still fulfilled the diagnostic criteria of DD 4 years later. On the neural level, children with DD showed reduced gray and white matter volumes in various regions and prominent tracts of the frontoparietal numerical network. These differences do not vanish over time, but persist from childhood into adolescence.

The dyscalculics showed reduced gray matter volumes in the parietal lobes specifically, but also in the occipital, temporal, and frontal parts of the brain, consistent with the results of previous studies. Less gray matter volume in the IPL including the IPS and the SPL have been reported in all studies with dyscalculic children (Rotzer et al., 2008; Rykhlevskaia et al., 2009; Ranpura et al., 2013). These regions are known from functional studies to be the key areas for number processing and quantity representation (for a meta-analysis see Sokolowski et al., 2017; Arsalidou et al., 2018). Furthermore, we also found reduced gray matter volumes in the bilateral supramarginal gyri, which are thought to play a crucial role in the retrieval of arithmetical facts (Menon, 2016). Similar to Rykhlevskaia et al. (2009), our results revealed lower gray matter volumes in the MOG/SOG, the cuneus/precuneus, and the temporal gyrus, although our results include bilateral MTG/ITG. Unlike the studies conducted before with DD children (Rotzer et al., 2008; Rykhlevskaia et al., 2009; Ranpura et al., 2013), we found reduced gray matter volumes in the insula. However, the insula has a high likelihood to be activated when children solve number and calculation tasks and have been proposed to play a role in intrinsic motivation about learning and training (Arsalidou et al., 2018). Also, our study did not find any volumetric differences in the parahippocampal areas, which was reported in Rykhlevskaia et al. (2009).

In terms of white matter, our study revealed reduced white matter volume in dyscalculics in the bilateral superior and ILF, the CST, the IFOF, and the ATR. Reduced white matter volumes were also reported in the same tracts by Rykhlevskaia et al. (2009), except that they found additionally reduced volumes in the forceps major and the splenium of the corpus callosum. Moreover, the CST, the ILF and SLF, and the corona radiata (of which the ATR is part) have all been associated with numerical and mathematical processing by numerous studies (Kucian et al., 2013; for a review see also Matejko and Ansari, 2015). The SLF connects frontal and parietal regions of the brain, which are known to be the main areas activated when solving number and arithmetic related tasks. It has further been proposed that the ILF is involved in visual processing related to numerical or mathematical problem solving (van Eimeren et al., 2008; Matejko and Ansari, 2015).

Children with DD revealed increased volumes in the right CST. This tract connects the cortex with the brainstem and is typically associated with motor functions. Research has further demonstrated that there is a link between finger and number representation (Noël, 2005; Matejko and Ansari, 2015). As children with DD often rely on finger counting strategies to compensate for the deficits in fact retrieval, the increased volume in the CST might be related to the frequent finger use during calculation in DD.

However, it is important to note that these observed structural differences in gray and white matter are related to underlying microstructural mechanisms of development and learning. Among the candidate mechanisms explaining gray and white matter plasticity are morphometric changes in the neuron (e.g., axon sprouting, dendritic branching, synaptogenesis, neurogenesis), in fiber organization (e.g., axon branching, sprouting, axon diameter or the number of axons) as well as in the myelination (for detailed information see Zatorre et al., 2012). But also vascular changes (angiogenesis) or changes in morphology and number of glia and astrocytes could explain the increase in gray and white matter volume (Zatorre et al., 2012). Regarding the structural differences in DD children, one could speculate that DD children show reduced white and gray matter volumes as the underlying microstructural process does not take place to the same extent as in TD peers. This would also be in line with results of functional and DTI studies reporting decreased activation and lower FA values in number related areas and tracks, respectively (Davis et al., 2009; Kucian et al., 2013; for an overview see Peters and De Smedt, 2018).

**Figure 2:**
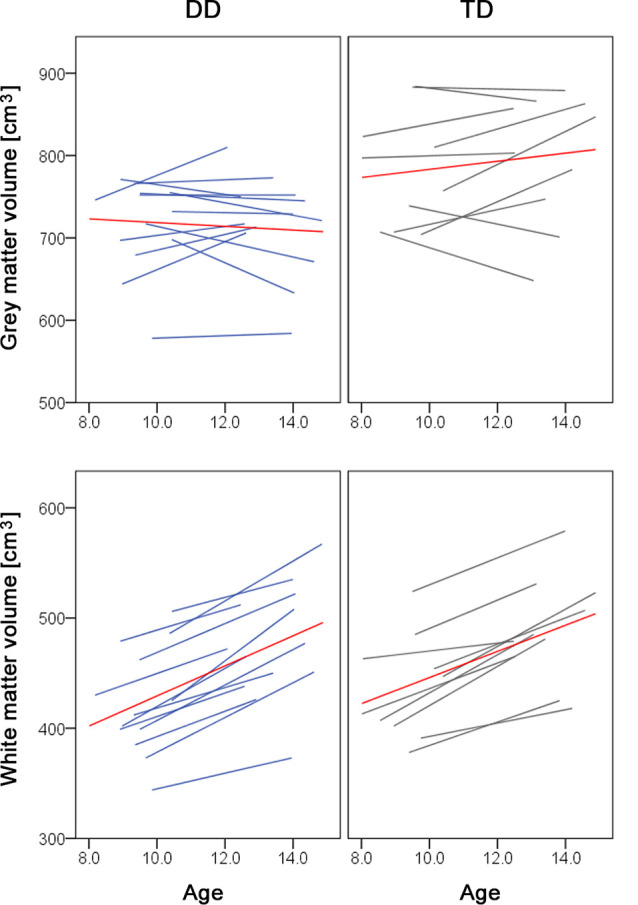
Spaghetti plots of whole brain volumes. Gray matter (top panel) and white matter volumes (lower panel) are plotted against age for children with dyscalculia (left, in blue) and typically developing children (right, in gray). The red line depicts the mean of the group.

Over development, we did not observe a gray matter decrease or prominent white matter increases. The lack of a gray matter decrease can be due to the age of our subjects. We examined children between the ages of 9 and 14. The developmental trajectory of the gray matter volume follows an inverted u-shape and depending on the study the peak of gray matter volume has been reported at age 8 (Mills et al., 2016), age 12 (Groeschel et al., 2010), or between ages 11–14 (Gogtay and Thompson, 2010). The time point of the peak further varies depending on the brain region (Giedd et al., 1999) and the sex and/or pubertal stage, with females reaching gray matter peaks 1–2 years earlier than males (Gogtay and Thompson, 2010; Mills and Tamnes, 2014). Therefore, it could be the case that we did not detect developmental changes in the gray matter, because the children and adolescents we studied are around one of the reported gray matter peaks. If we look at the individual trajectories of our study participants, some children still show a gray matter increase, whilst other children show a gray matter decrease or almost no change in the gray matter volume over the 4 years (Figure 2, upper panel). Moreover, the fact that we controlled for pubertal status and therefore indirectly for sex might be an additional reason why we do not find developmental changes. In the white matter volume, we found a significant increase during development in the left SLF (MNI *x* = −40, *y* = −20, *z* = 28), located right next to the reported developmental changes in the study of Giorgio et al. (2010; MNI *x* = −42, *y* = −22, *z* = 28). However, our results do not show the prominent white matter changes as reported in the literature (Giorgio et al., 2010). A possible explanation is that our study does not look at the correlations between white matter volume and age. On the other hand, the individual trajectories of our subjects show a clear increase in the total white matter volume (Figure 2, lower panel), which is in line with previous research (Aubert-Broche et al., 2013; Mills et al., 2016).

The main focus of our study was to find out more about the developmental trajectories of gray and white matter volumes in children with DD. We found no significant interactions, but the group differences in gray and white matter volumes remained stable during the examined time window. Although the differences found between DD and TD children are well in line with the literature (Rotzer et al., 2008; Rykhlevskaia et al., 2009), and also reflect the known and persistent behavioral differences (Nelson and Powell, 2018), the developmental trajectories differ from the results reported by Ranpura et al. (2013) in such that we did not find an increase in white matter volume in frontal and parietal areas in TD children only. Studies in adults find no or very subtle differences between the subjects with and without DD (Cappelletti and Price, 2013; Moreau et al., 2019). Based on these results, one could argue that the volumetric differences should diminish over development. However, Moreau et al. (2019) used a rather lenient criterion for DD, which could also be a reason why volumetric differences were not found. Also, there is an age gap of 10–15 years between our results and the adult studies, in which developmental changes can still take place. Our results, therefore, point towards stable and persistent differences in the dyscalculics gray and white matter volumes from childhood to adolescence. More research, specifically longitudinal studies over a longer time interval, are urgently needed to enable us to conclude the developmental trajectory of the brain structure in DD. However, findings from the present study suggest that development proceeds in a similar manner between DD and TD children.

To better understanding the present findings, it is important that we advance our knowledge about the typical developmental trajectory of the brain structure and its spontaneous variations in numerical cognition. Furthermore, the effects of schooling and specific interventions on brain structure need to be explored more profoundly. This knowledge should build the basis for the investigation and a better understanding of the deviant and/or delayed development as reported in children with DD. In a next step, it would be interesting to investigate if the abnormalities in gray and white matter are a result of a developmental delay or a specific marker of DD. This open question could be tackled by comparing the structural brain development of DD children with TD children that perform on a similar numerical level. Furthermore, the investigation of structural changes caused by a specific intervention could help clarify the questions about the neurobiological cause of dyscalculia.

### Limitations

Our results are important for the field since this is to our knowledge the first study investigating the developmental trajectory of structural white and gray matter volumes using longitudinal data in DD. However, several limitations should be considered when interpreting the results of our study. First of all, due to braces and movement artifacts, the drop-out rates in longitudinal MRI studies with children and adolescents are high. Our study includes only small sample sizes and should for this reason be interpreted with caution. However, the data included had good data quality ratings, as assessed by objective criteria (Gaser and Dahnke, 2016). Moreover, we replicated the main findings of previous studies examining volumetric gray and white matter differences in DD children (Rotzer et al., 2008; Rykhlevskaia et al., 2009; Ranpura et al., 2013), which were also performed with larger sample sizes (e.g., Rykhlevskaia et al., 2009). Therefore, we are confident that despite the small sample sizes our results contribute valuable knowledge towards an understanding of the developmental trajectory of brain structure in children with and without DD.

Second, the rather large age range within our sample (at each time point) may attenuate the developmental effects. For methodological reasons, a narrower age range would be much better to examine general developmental effects and detect group differences over time. A closer look at our data revealed that the individual developmental trajectories showed similar trends irrespective of the age at the entry of the study. However, future longitudinal studies should investigate limited age ranges to control better for the effect of schooling and the rapid changes in development.

A third restricting point of our study is the significant difference in IQ between the TD and the DD group. Developmental imaging studies have shown that there is a positive relationship between intellectual abilities and white/gray matter volume, especially in the dorsolateral prefrontal cortex, parietal lobe, the anterior cingulate cortex and in temporal and occipital regions (Wilke et al., 2003; Tamnes et al., 2011; Brancucci, 2012). For this reason, we ran our analyses with IQ as an additional covariate showing that the main results remained unchanged (see Supplementary Material).

Last, it should be noted that some authors argue that the sensitivity to examine the white matter using voxel-based morphometry is limited, as white matter areas are characterized by large homogenous regions with only subtle changes in intensity (Kurth et al., 2015).

## Conclusion

In conclusion, the present study reveals for the first time the gray and white matter trajectories of the dyscalculic brain. The findings confirm the structural differences as reported in earlier research and support the notion that DD is characterized by persistent structural and behavioral abnormalities. There is an urgent need for longitudinal studies examining the typical and atypical neural development, but also the effect of interventions and therapy on numerical and mathematical abilities. Advancing the knowledge about the developmental course of DD and the effects of schooling, therapy, and intervention would enable us to support affected children and adolescents more effectively.

## Data Availability Statement

The datasets generated for this study will not be made publicly available. According to the Ethics committee of Zurich, Switzerland the consent was not obtained for sharing data outside the research team. Therefore we do not have the allowance to make the data publicly available. Requests to access the datasets should be directed to the Ethics committee of Zurich in Switzerland (Info.KEK@kek.zh.ch, project-number: 2011-0384, title of the project: Plasticity after training and development in children with and without dyscalculia).

## Ethics Statement

The present study was reviewed and approved by the Ethics committee of Canton of Zurich, Switzerland. Written informed consent to participate in this study was provided by the participants’ legal guardian/next of kin.

## Author Contributions

All authors have contributed and have approved the final manuscript. UM contributed to the design of the study, the acquisition, analysis, interpretation of the data, and writing the manuscript. MA and RO’G contributed to data interpretation and revised the manuscript. KK contributed to the design of the study, the acquisition, data interpretation, editing, and revision of the manuscript.

## Conflict of Interest

The authors declare that the research was conducted in the absence of any commercial or financial relationships that could be construed as a potential conflict of interest.
