# Supplementary material for: Persistent Differences in Brain Structure in Developmental Dyscalculia: A Longitudinal Morphometry Study
Source: Front Hum Neurosci. 2020 Jul 17;14:272. doi: 10.3389/fnhum.2020.00272 (PMC7379856; doi:10.3389/fnhum.2020.00272)
Supplement: Supplementary file 1 [file Data_Sheet_1.docx]

Kanton Zürich
Kantonale Ethikkommission

Prof. Dr. med. Peter Meier-Abt
Präsident

Niklaus Herzog, lic. iur. et theol.
Juristischer Sekretär
Stampfenbachstrasse 121
Postfach
8090 Zürich
Telefon +41 43 259 79 70
Fax +41 43 259 79 72
eliane.sueess@kaz.zh.ch
www.kek.zh.ch

Universitäts-Kinderspital Zürich
PD Dr. sc. nat. Karin Kucian
Steinwiesstrasse 75
8032 Zürich

Zurich, 4 August 2015

KEK-ZH-Nr. 2011-0384
Title of Project:
Plasticity after training and development in children with and without dyscalculia

Dear Dr Kucian

For the project (Plasticity after training and development in children with and without dyscalculia) of which the present study is a part, consent was not obtained for the sharing of data outside the research team. It is therefore not possible to make the data publicly available, even in a pseudo-anonymised (coded) form. If researchers are interested in accessing the data, they can apply to the KEK Zurich and their application will be assessed on a case by case basis, possibly requiring additional ethics approval and/or informed consent from the participants.

Sincerely yours

Niklaus Herzog

Niklaus Herzog
